# Studying the Impact of the DDB2 T338M Missense Mutation on the Development of Equine Squamous Cell Carcinoma and Sarcoid

**DOI:** 10.3390/ani15070911

**Published:** 2025-03-22

**Authors:** Hannah Quatember, Barbara Nell, Barbara Richter, Doris Rigler, Marlies Dolezal, Sabine Sykora, Barbara Wallner

**Affiliations:** 1 Department for Small Animals and Horses, Clinical Centre for Equine Health and Research, University of Veterinary Medicine Vienna, Veterinärplatz 1, 1210 Vienna, Austria; hannah.quatember@vetmeduni.ac.at (H.Q.); barbara.nell@vetmeduni.ac.at (B.N.); sabine.sykora@vetmeduni.ac.at (S.S.); 2 Department of Biomedical Sciences and Pathobiology, Institute of Pathology, University of Veterinary Medicine Vienna, Veterinärplatz 1, 1210 Vienna, Austria; barbara.richter@vetmeduni.ac.at; 3 Department of Biomedical Sciences and Pathobiology, Animal Breeding and Genetics, University of Veterinary Medicine Vienna, Veterinärplatz 1, 1210 Vienna, Austria; doris.rigler@vetmeduni.ac.at; 4 Department of Biomedical Sciences and Pathobiology, Platform for Bioinformatics and Biostatistics, University of Veterinary Medicine Vienna, Veterinärplatz 1, 1210 Vienna, Austria; marlies.dolezal@vetmeduni.ac.at

**Keywords:** Haflinger, cancer, eye, tumour, genetic risk factor, DDB2, squamous cell carcinoma, equine sarcoid

## Abstract

A missense mutation in damage-specific DNA binding protein 2 (DDB2 c.1013 C>T; p.Thr338Met) has been described as a risk factor for ocular squamous cell carcinoma (OSCC) in the Haflinger breed. Here, we examined the impact of DDB2 C>T allele status on the development of OSCC, squamous cell carcinoma (SCC) at other localisations, or equine sarcoid (ES) in Haflingers and other breeds with a high incidence of these tumour types. We genotyped affected Haflinger, Noriker, Warmblood, and Icelandic horses. Results based on 56 Haflingers confirmed the significantly higher risk for OSCC in DDB2-TT Haflingers but also suggested an increased risk in heterozygous (DDB2-CT) Haflingers. We also found the DDB2-T allele in Norikers with OSCC but not in Warmbloods. Only one homozygous DDB2-T allele carrier was among the 23 Haflinger and 44 Noriker/Warmblood/Icelandic horses with an SCC at a localisation other than the eye or ES. Overall, our data underline the severity of the DDB2-T allele with regard to OSCC and provide no evidence for the impact of the DDB2 risk allele status on the development of ES and SCC at localisations other than the eye.

## Simple Summary

Squamous cell carcinoma (SCC) and equine sarcoid (ES) are the most common tumours in horses. It has been shown previously that Haflinger horses with a homozygous genotype for a missense variant in the DDB2 (damage-specific DNA binding protein 2) gene have an increased risk of developing ocular SCC (OSCC). Genetic testing for this variant is commercially available. Here, we studied the DDB2 genotype in patients with SCC, OSCC, or ES at the Vetmeduni Vienna. Results confirmed the impact of the DDB2 risk variant on the development of OSCC in Haflingers. We found homozygous risk allele carriers in OSCC patients of the Noriker breed, but we did not detect the risk allele in Warmbloods with OSCC. No evidence of an influence of the DDB2 genotype on the development of ES and squamous cell carcinoma in sites other than the eye was found. Our results underline the impact of the DDB2 risk allele on animal health. In Haflingers, the DDB2 genotype should be considered in mating strategies and preventive management. Furthermore, regular veterinary eye examinations for early detection of OSCC are recommended in this breed.

## 1. Introduction

Squamous cell carcinoma (SCC) and equine sarcoid (ES) are the most common cutaneous neoplasms in horses. SCCs predominantly develop at mucocutaneous junctions of the oral or nasal cavity (head and neck), the genital tract, and the eye [1,2]. Ocular squamous cell carcinomas (OSCC) are primarily detected at the limbus and the nictitating membrane [3,4,5,6]; they grow locally invasive and have a low rate of metastasis (0–18.6%) [7,8,9,10,11,12].

The etiopathogenesis of OSCC is multifactorial—primarily, exposure to ultraviolet (UV) radiation and a lack of photoprotective pigmentation contribute to increased risk, as both promote DNA damage, leading to uncontrolled cell growth and tumour development [5]. Horses with a lack of periocular pigment [13] or horses expressing only pheomelanin, which is the case in chestnut coat colour, are at increased risk of developing OSCC compared to horses with eumelanin-based colours, such as bay and black [4]. It has also been suggested that a deficiency in androgens and oestrogens promotes the development of OSCC, as geldings appear to be affected more often than stallions or mares [14,15,16]. OSCCs develop in adult horses, and affected animals are usually older than 11 years [4,7,17]. Without proper treatment, OSCCs can cause visual impairment and lead to the loss of the eye. OSCCs can extend into orbital tissues and the underlying bone and become a common reason horses need to be euthanised. Various treatment options have been described [7,9,11,17,18,19], but the recurrence rate is remarkable (14.3–16.7%) even when surgical excision is performed with a laser [11,20]. While equine papillomavirus (EcPV) plays a significant role in the aetiology of SCC in localisations other than the eye [13,21,22,23,24] and bovine papillomavirus (BPV) in ES [25,26,27,28,29], their involvement in OSCC is still under debate [13,21,22,30].

There is a hereditary predisposition to OSCC, and the breeds with the highest incidence described so far are the Appaloosa, the American Paint Horse, and the Haflinger [5,12,16]. A recessive inheritance pattern has been suggested for the increased risk of OSCC in Haflingers [5]. In 2017, a missense variant in exon 7 of the damage-specific DNA binding protein 2 gene on chromosome 12 (DDB2, c.1012 C>T, p.Thr338Met) has been described to be associated with increased risk for limbal and third eyelid SCC in this breed [12]. The intact DDB2 protein is required for the recognition and repair of UV-damaged DNA [31]. The mutated T338Met mutant protein cannot efficiently bind to UV-damaged DNA, and this results in an impaired repair mechanism, linking genetic and environmental risks [12,32]. The manifestation of the DDB2-T allele has been described as recessive, and homozygous DDB2-TT allele carriers explained about 76% of the Haflinger OSCC cases. The DDB2 genotype is, therefore, strongly associated but not perfectly concordant with the OSCC status. Moreover, Haflinger horses homozygous for the DDB2 risk allele (TT) are suggested to have 5.89 times the risk of developing OSCC than horses with genotypes CT or CC [12].

The DDB2-T allele segregates at a high frequency in the Haflinger breed (25%) [12], and it also has been detected in draft horses (Belgian, Clydesdale, Shire), Rocky Mountain Horses, Holsteiner, Belgian Warmbloods, and Connemara Ponies [12,32,33]. Consideration of the DDB2 genotype in mating strategies is recommended for Haflingers, as is intensive veterinary monitoring in homozygous “high-risk” horses [34].

Genetic testing for disease-reported variants is becoming more and more important in domestic animals. Genotyping results can affect treatment and prognosis, alter the value of an individual, and build the base for mating decisions, which affects the genetic composition of the breed in the next generations. Recently, an elevated frequency of disease- and trait-associated variants has been shown in horses [35], and some disease-causing variants are unlikely to be contributing to disease in a simple Mendelian inheritance pattern. Therefore, extensive validation of the clinical consequences of putative disease-causing variants, also across breeds, is needed.

The influence of the homozygous DDB2-T allele on OSCC in Haflingers has been demonstrated and independently confirmed in American and European Haflingers [16,36], but there is still uncertainty as to what extent the DDB2 genotype contributes to the occurrence of (i) OSCC in other breeds and (ii) SCC at locations other than the eye. So far, it has not been studied whether the DDB2 risk allele also impacts the development of the most common skin tumour in horses, the equine sarcoid (ES). While it is generally accepted that infection with BPV is necessary for the development of ES [26], a genetic predisposition of the host is also suspected [27,28,29]. In some breeds, an association of certain equine leukocyte antigen (ELA) haplotypes and ES has been shown [37,38,39,40], and various familial, coat colour, as well as breed-related predispositions have been described [41,42,43,44]. According to recent studies, a polygenic architecture with multiple unlinked susceptibility loci involved seems to best describe the genetic basis [27,28,29]. The DDB2 gene was not yet among the loci associated with ES, as this protein is predominantly linked to the repair of ultraviolet light-damaged DNA [31]. But, very recent studies in humans suggest additional non-canonical roles of DDB2 [45,46,47] and provide evidence for its involvement in the aetiology of different cancer types. The Haflinger, a breed with a high frequency of the mutated DDB2 allele [12], is an ideal population for investigating the putative impact of the DDB2 allele status on the development of ES.

In this study, we address the impact of the DDB2 allele on the development of OSCCs, SCCs, and ESs by evaluating DDB2 C>T genotypes from histopathologically confirmed patients. We first determined the breeds with the highest incidence of the respective diseases via an extensive database search of patients at the University of Veterinary Medicine (Vetmeduni) Vienna presented in the past 20 years. We then focused primarily on two common Austrian breeds, the Haflinger and the Noriker, but also included breeds whose DDB2-genotype had not been investigated before (Icelandic horses) or the segregation of the DDB2-T was expected from the previous literature (Warmblood). Our results, based on archived biological material from patients, confirmed and extended knowledge on the association of the DDB2 allele status and OSCCs in Haflingers, revealed another breed carrying the risk allele, and provided no evidence for the involvement of the DDB2 variant on genital/head and neck SCCs or ESs.

## 2. Materials and Methods

### 2.1. Database Search

The database system ‘Tierinformationssystem’ (TIS) of the University of Veterinary Medicine in Vienna was searched to determine the breed distribution of horses presented at the Equine Surgery Unit or the Ophthalmology Unit of the Clinical Centre for Equine Health and Research of the Vetmeduni Vienna. We extracted patients in a 20-year period (2002–2022) with diseases that were treated. For this analysis, we excluded healthy horses that were presented, for example, for castration, pre-purchase examination, or as companion horses for patients. The remaining horses were only included once with their chronologically first diagnosis listed – dental, orthopaedic, ophthalmic, oncological, colic, all elective, ambulatory and emergency cases, and all elective and emergency surgeries. Next, the database was filtered for horses diagnosed with SCC or ES in the given period. For these horses, sex, age at first presentation at the clinic, and tumour localisation for SCCs (eye, head, neck, or genital) were collected, if available. The tumour localisation of ‘head and neck’ included extraorbital structures such as the sinus system, nostrils, alveoli and nasal, and pharyngeal and oral cavities.

### 2.2. Sample Collection

A total of 144 horses with a histopathologically confirmed diagnosis of either OSCC, SCC at other localisation (genital, head and neck region), or ES were selected for sample collection. We focused on four breeds with a high incidence of these tumours. Based on our results (Section 3.1), we considered purebred Haflinger, Noriker, Warmblood, and Icelandic horses. Either hair root material from the mane (18 horses) or formalin-fixed paraffin-embedded (FFPE) tissue (126 horses), archived at the Institute of Pathology at the Vetmeduni Vienna, was collected. FFPE material originated either from the tumour or from another organ of the patient. For the 144 horses, we compiled information on the breed, sex, coat colour, tumour location, histopathological diagnosis, and age at first diagnosis of the tumour at the Vetmeduni.

For the OSCC-free control group, the horses had to meet the following criteria: a registered Haflinger, at least thirteen years old (this is one standard deviation above the mean age of diagnosis for limbal SCC in the Haflinger breed) [5] and free of any ocular lesions or suspicious tumour growth anywhere on the body. The latter information was obtained either from the TIS database system, or a clinical investigation of animals, including an eye examination with a particular focus on the cornea, conjunctiva, nictitating membrane, and eyelids. The clinical examination was conducted by a veterinarian trained by an ECVO Diplomate. Hair roots (*n* = 22) or archived FFPE tissue (*n* = 36) were collected from the control horses. Details on all samples are given in Supplementary Table S1.

### 2.3. Genotyping

Genomic DNA (gDNA) was extracted from approximately 30 hair bulbs per animal using the DNeasy Blood and Tissue Kit (Qiagen ^®^, Hilden, Germany). For gDNA isolation FFPE tissue, 8 to 10 slices with a thickness of 10 µm were cut from the tissue block using a microtome. gDNA was isolated with the isolation kit ReliaPrep™ FFPE gDNA Miniprep System (Promega, Madison, Wisconsin) following the manufacturer’s protocols. DNA quantity and integrity were assessed by loading 5 µL of DNA solutions onto a 0.8% agarose gel. gDNA was then adjusted to a concentration of 5–10 ng/µL using TE-Buffer.

For allele determination at the DDB2 risk allele locus (OMIA 000735-9796, NC_009155.3: g.11726667C>T, c.1013C>T, p.(T338M), rs1139682898) a PCR-based KASP™ (Kompetitive Allele-Specific PCR) genotyping assay was designed (KASP™, lgcgroup.com (accessed on 15 January 2025). KASP™ screening was performed on a CFX96 Touch™ Real-Time PCR Detection System using the standard protocol provided by the supplier (LGC, Berlin, Germany). Each run included six positive controls with known allelic status (two homozygous CC, two heterozygous CT and two homozygous TT) and two no-template controls (NTCs). Cluster plots were analysed using Bio-Rad CFX Manager 3.1 (Biorad, Vienna, Austria).

### 2.4. DNA Uracil-DNA Glycosylase (UDG) Treatment

Fixation of sample material in formalin, as is performed in FFPE material, can cause DNA damage through cytosine to uracil deamination, which may alter the genotype determined from FFPE samples by detecting cytosines as thymines [48]. To evaluate whether the deamination of cytosine to thymine (C>T) in DNA isolated from FFPE tissue resulted in genotyping artefacts, 61 DNAs isolated from FFPE tissue (samples listed in Supplementary Table S1) were treated with uracil DNA glycosylase [49]. For this purpose, 20 ng of gDNA were mixed with 2 μL 10 x UDG Buffer (New England Biolabs, Ipswich, Massachusetts) and 0.2 μL (1U) UDG (M0280S, New England Biolabs, Ipswich, Massachusetts) in a volume of 20 µL and incubated for 2 h at 37 °C. The enzyme was deactivated at 95 °C for 10 min. KASP™ genotyping was performed for the DDB2 risk allele, using the UDG-treated DNA as a template, as described above.

### 2.5. Statistical Analysis

All statistical data analysis was performed with R version 4.2.2 [50,51] and R studio Version 2023.06.1+524. Data were prepared for analysis using functions from a package tidyverse [50,51]. A Fisher’s Exact test (fisher.test) was carried out to calculate odds ratios for the largest breed groups representing patients diagnosed with SCC, OSCC, or ES.

Pearson’s Chi-squared test with Yates’ continuity correction (chisq.test) was performed to determine if there was a significant difference in the number of geldings and stallions/mares between Haflinger and Warmblood OSCC patients.

We tested for association of DDB2 genotypes to OSCC in 56 Haflinger cases and 58 controls, using logistic regressions with logit link function, applying function glm (family = ”binomial”) assuming either (1) additive inheritance, modelling the number of minor alleles (T) as covariate, (2) recessive-dominant inheritance, or (3) allowing for all possible modes of inheritance by fitting DDB2 genotype as a fixed categorical effect.

Significance is declared at an alpha of 5% after multiple testing corrections with the Tukey HSD method [52], considering a multiple testing load of three contrasts for model 3.

Estimated marginal means for each genotype and pairwise differences between them were calculated using function emmeans (option pairwise) in package emmeans v1.10.5 [53] and then back-transformed to probabilities (option type = ‘response’). Results are shown as bar plots produced with packages RColorBrewer v1.1-3 [54], ggplot2 v3.5.1 [55], and ggpubr v0.6.0 [56]. The height of the bars corresponds to estimated marginal means, and the whiskers depict the upper and lower 95% confidence intervals. Pairwise contrasts are indicated by *p*-value brackets.

We then used hierarchical model comparisons applying function Anova between models 1–3 and 2–3, respectively, to identify the best-fitting genetic inheritance pattern. We note that hierarchical model comparison between models 1 and 2 was not possible because both have the same number of degrees of freedom.

We verified that our Haflinger control animals were, indeed, significantly older than our OSCC cases with a linear model on log10 transformed age in years and a fixed categorical effect of case vs. control group. We tested if the DDB2 genotype affected the age of the horses when presented to the clinics with a linear model on log10 transformed age in years and a fixed categorical effect of the genotypes.

## 3. Results

### 3.1. Database Search on Patients Presented to the Clinic

The spectrum of horse breeds presented at the Equine Surgery Unit or the Ophthalmology Unit of the Clinical Centre for Equine Health and Research of the Vetmeduni Vienna was evaluated from 14,186 patients treated between 2002 and 2022.

**Figure 1:**
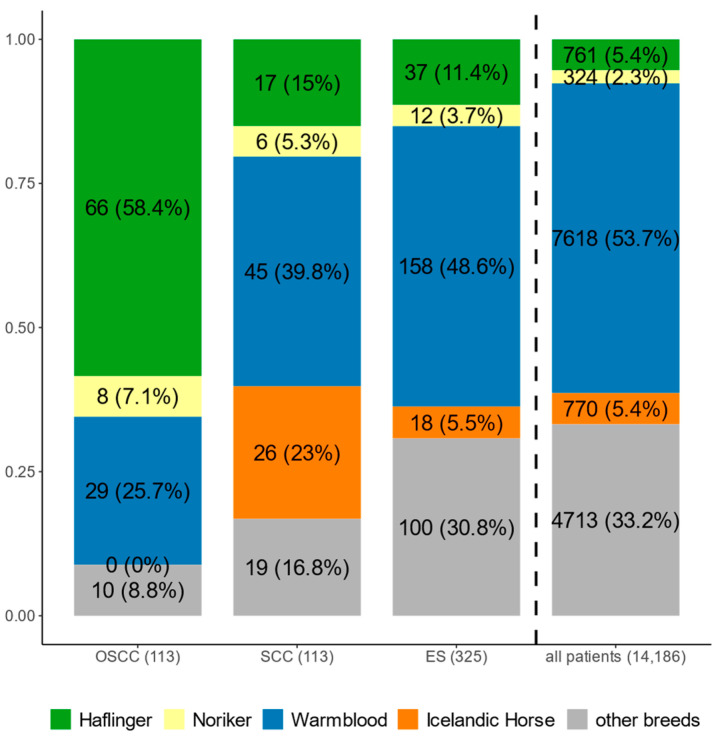
Breeds presented to the Centre for Equine Health and Research of the Vetmeduni Vienna in a period of 20 years (2002–2022). Given is the number of horses from different breeds diagnosed with OSCC, SCC at other localisation, or ES (details in Section 2.1), followed by the number of all patients. Number of patients is indicated, and the percentage is given in parentheses. Detailed information on ‘other breeds’ is provided in Supplementary Table S2.

More than half of the patients (7618 horses; 53.70%) were Warmbloods, and 5.36% (761 horses) were Haflingers. Out of those, 113 horses were diagnosed with an OSCC, and 113 had an SCC on another localisation than the eye. Three-hundred-twenty-five horses were diagnosed with ESs. Figure 1 shows the breed distribution among the different groups, and details are given in Supplementary Table S2. Among the 113 horses with OSCC, Haflingers represented the largest proportion, with 66 horses (58.40%), followed by Warmbloods (29 horses; 25.66%). Additionally, eight Norikers (7.08%) and ten horses from the diverse group of ‘other breeds’ (8.85 %) were diagnosed with OSCC, but no Icelandic horse. The picture changed when focusing on SCCs at other localisation, which included genital SCCs and SCCs in the head and neck region (*n* = 113). Here, Warmbloods formed the largest group with 45 horses (39.82%), followed by 26 Icelandic horses (23.01%), the group of ‘other breeds’ (19 horses, 16.81%), and Haflingers in fourth place with 17 horses (15.04%). It is worth mentioning that Icelandic horses were mainly diagnosed with genital SCCs. The largest groups among the 325 horses diagnosed with ES were Warmbloods, with 158 horses (48.62%), and the group of ‘other breeds’, with 100 horses (30.77%), followed by 37 Haflingers (11.38%), 18 Icelandic horses (5.54%), and 12 Norikers (3.69%).

The high incidence of OSCC in the Haflinger and Noriker breeds was evident from their overrepresentation at the clinic and resulted in an odds ratio (OR) of 27 and 3.37, respectively. SCCs at other localisations were also frequently present in Haflingers (OR = 3.17) and particularly in Icelandic horses (OR = 5.35) (Figure 1). The breed composition in the 325 horses with ES followed the frequency of breeds represented at the Equine Surgery Unit of the Centre for Equine Health and Research, with a slight increase in Haflingers (OR = 2.33) and Norikers (OR = 1.65). Overall, results confirmed the high incidence of OSCC in the Haflinger breed and revealed a predisposition for OSCC in the Noriker breed, as well as SCC and ES in Haflingers.

Among OSCC cases at the clinic, geldings were significantly overrepresented in the 29 Warmbloods (24 geldings, 5 mares) but not in the 66 Haflingers (31 geldings, 2 stallions, 33 mares), *p* = 0.0025. Furthermore, the age of presentation to the clinic in this cohort was slightly higher in Warmbloods (median = 17.70, range 4–26 years) than in Haflingers (median = 12.01, range 4–32 years).

### 3.2. DDB2 Risk Allele Status in Horses Diagnosed with OSCC, SCC and ES

We focused on the breeds with the highest incidence of OSCC, SCC, or ES (Haflinger, Noriker, Icelandic horse and Warmblood) and collected tissue from affected horses to determine the DDB2 genotype by genotyping. For OSCC, we analysed 56 Haflingers, 7 Norikers, and 14 Warmbloods; the SCC group included 12 Haflingers, 1 Noriker, 26 Warmbloods, and 13 Icelandic horses. Eleven Haflingers and four Norikers formed the sarcoid group. Additionally, a Haflinger tumour-free control group was designed (*n* = 58). Sample details are given in Section 2.2 and Supplementary Table S1.

Hair root or archived FFPE tissues were used, and the FFPE material had been stored between 0.5 and 19 years. Fixation of sample material in formalin often leads to DNA damage as cytosine deaminates to uracil [49], and this could have distorted our genotyping results. Therefore, we digested 61 genomic DNAs isolated from FFPE material with uracil-DNA glycosylase (UDG) to reduce such C>T artefacts [48]. The DDB2 genotype before and after UDG treatment perfectly matched in all analysed samples (Table 1 and Supplementary Table S1), proving the validity of the DDB2 genotypes generated from DNA isolated from FFPE archived material.

We detected the DDB2-T allele in the Haflinger and Noriker breeds but not in Warmbloods and Icelandic horses. An overview of genotyping results is given in Table 2.

#### Influence of the DDB2 Risk Allele on the Development of OSCC in Haflingers

**Figure 2:**
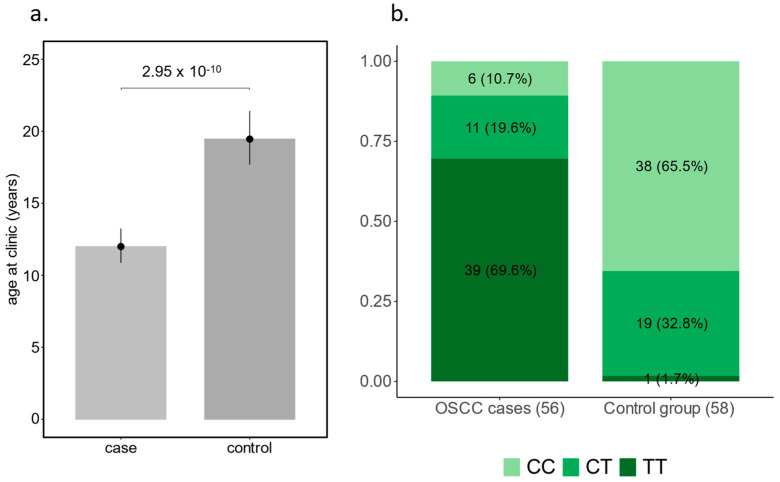
56 Haflinger OSCC cases and 58 tumour-free matched controls. (**a**) shows age of cases and controls at investigation; (**b**) displays numbers and proportions of DBB2 genotypes.

We compared DDB2 genotypes of 56 Haflingers with OSCC with 58 tumour-free Haflingers as a control group. In the Haflinger OSCC group, 39 (69.6%) were homozygous for the DDB2 risk allele (TT), 11 (19.6%) were heterozygous (CT), and only 6 (10.7%) were homozygous wildtype (CC). In the control group, only one Haflinger (1.7%) was homozygous for the DDB2 risk allele, 19 were heterozygous (32.8%), and with 28 (65.5%), the majority was homozygous wildtype (Figure 2). T-allele frequency was estimated at 79.5% in the diseased horses and 18.1% in the control group, respectively.

**Figure 3:**
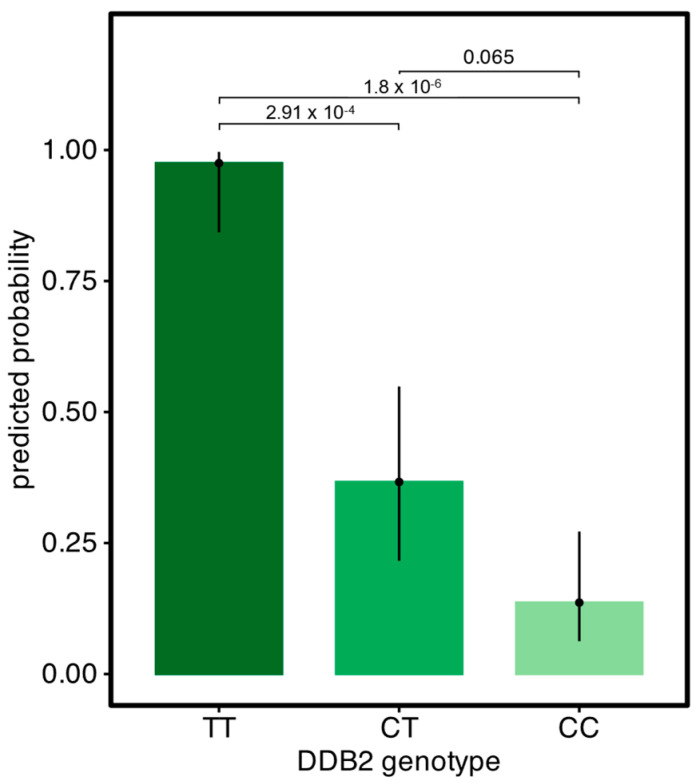
Predicted probability for developing an OSSC for the three DDB2 genotypes based on 56 Haflinger cases and 58 matched tumour-free controls with *p*-values for the contrasts.

When testing the association between the DDB2 genotype and OSCC status, the model best fitting the data was with the DDB2 genotype as a fixed categorical effect (model 3). As shown in Figure 3, the predicted probability for developing an OSCC is significantly higher in DDB2-TTgenotypes than in DDB2-CC or CT (*p*-value < 0.05), while the effect in heterozygotes is visible but not significant with our chosen threshold (*p*-value: 0.065). However, the confidence intervals for the estimation of marginal means, which reflect the number of observations available, suggest the reduced power due to the limited number of observations for CC and CT horses. An odds ratio of 247 for homozygous TT allele versus CC highlights the impact of the DDB2 genotype on OSCC, while an odds ratio of 3.67 for CT versus CC also indicates an increased risk in the heterozygous state. Accordingly, the estimated relative risk for developing an OSCC compared to CC was 2.69 for CT and 7.15 for TT genotype Haflingers.

**Figure 4:**
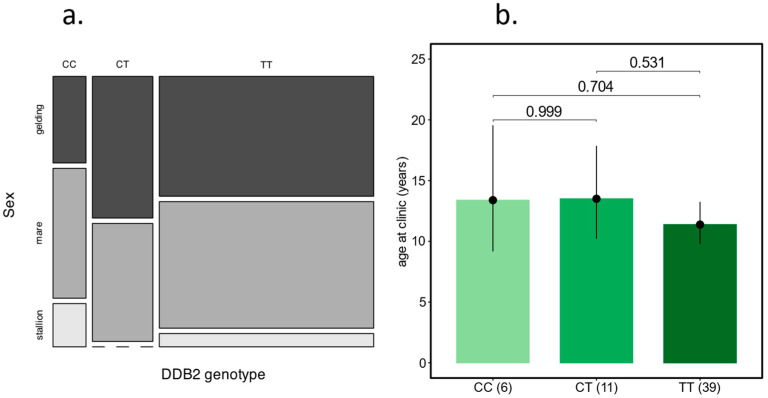
DDB2 genotype in Haflingers and the sex and age of OSCC diagnosis. (**a**) Mosaic plot showing the proportion of the sexes in six CC, eleven CT, and 39 TT cases. (**b**) Barplots of back-transformed estimated marginal means of age at presentation to the clinic. Data are given in Supplementary Table S1.

We observed no differences in the composition of the sexes among the genotypes in 56 Haflingers (Figure 4a). Haflingers with OSCC presented to the clinic are slightly younger than Warmbloods (Section 2.1). We tested 56 genotyped OSCC cases to determine whether the age at presentation to the clinic was affected by the DDB2 genotype. As shown in Figure 4b, the 39 DDB2 homozygous risk allele Haflinger (TT) in our dataset were not significantly younger than the six homozygous wildtype or the eleven heterozygotes.

The DDB2 genotype was also determined in seven Norikers and 14 Warmbloods diagnosed with OSCC (Table 1). While the DDB2 risk allele was not detected in any of the Warmbloods, four Norikers were tested homozygous TT, two were tested heterozygous, while only one Noriker was a homozygous wildtype. This indicates that the DDB2 risk allele could also pose a problem in the Noriker breed.

### 3.3. DDB2 Risk Allele in SCCs at Localisations Other than the Eye

Among the horses brought to the Equine Surgery Unit of the Centre for Equine Health and Research of the Vetmeduni Vienna, Haflinger, Noriker and Icelandic horses had a high incidence of SCC at localisations other than the eye (Figure 1). To investigate whether the DDB2 risk allele was involved in the development of these SCCs, a sample set of 52 horses was created, which had an SCC diagnosed at different localisations such as the head and neck (23) or the genital regions (29) (Section 2.2, Table 1, Supplementary Table S1). Only one Haflinger with an SCC in the head and neck group was homozygous TT. The 9-year-old DDB2-TT Haflinger gelding (case 111) was histopathologically diagnosed in 2014 with an SCC on the left lower lip. In addition, this horse had carcinoma in situ on the left nostril and a hyperkeratotic skin lesion on the front chest, which was later diagnosed as ES. Three months after the removal of lip and nostril tumours, another SCC without lymph node involvement was diagnosed in the left intermaxillary region. Interestingly, a plain depigmented area was detected in the temporal canthus of the right eye, which was not investigated further at that time. The horse died in 2015; the circumstances surrounding its death were not disclosed. Three horses, two Haflingers and one Warmblood, from the group of patients with SCC at localisations other than the eye were identified as heterozygous for the DDB2 risk allele. Our data do not provide evidence that the DDB2 risk allele is involved in the development of SCCs in localisations other than the eye.

### 3.4. DDB2 Risk Allele in ESs

Based on the fact that the DDB2 risk allele is present in the Haflinger and Noriker breeds, we investigated whether the DDB2 risk allele was also involved in the development of ESs. We genotyped eleven Haflingers and four Norikers diagnosed with ES and found that none of them were homozygous for the DDB2 risk allele. Only one Haflinger and two Norikers were heterozygous, indicating that there was no association between the DDB2 risk locus and the onset of ES.

## 4. Discussion

Seven years have passed since the DDB2-T allele was described to be associated with the increased incidence of OSCC in the Haflinger breed. Although the functional consequences of the mutated protein on the repair mechanism of UV-damaged DNA have been shown [31], the incomplete penetrance of the alleles and the coaction of other genetic (coat colour, sex) as well as environmental factors (UV light exposure, age) that contribute to disease progression, make it challenging to predict the impact of this single genomic variant mutation. But, knowledge of the effect of the genetic risk factor is important for responsible breeding and veterinary care.

The Haflinger is a very common breed in Austria, with more than 3000 registered mares, about 90 stallions and approximately 900–1000 foals born each year. All Haflinger sublines are represented in the Austrian population [57] and our dataset. About 5% of the horses treated at the Vetmeduni Vienna are Haflingers, and our database analysis clearly confirmed the high incidence of OSCCs but also showed a higher incidence of other SCCs and ESs in this breed. Based on archived material from patients in the past 20 years, we investigated the impact of the DDB2 risk allele on the development of OSCCs and SCCs at other localisations or ESs in Haflingers and also in three other breeds (Noriker, Warmbloods, and Icelandic horses). We conducted our retrospective study on gDNA from archived FFPE material.

In order to evaluate the association of the DDB2 C>T locus, 56 histopathologically confirmed Haflinger OSCC cases were analysed. The proportion of OSCC cases explained by homozygous DBB2 TT was lower in our dataset (69.6%) compared to the 76% detected in previous studies [12,16]. This could result from a probably broader genetic background represented by the horses in our dataset, as our samples covered the past 20 years, while members of a half-sibling family were included in [12,16].

We used a concisely defined control group of tumour-free Haflingers. The frequency of the T-allele was lower in our tumour-free control (18%) compared to previous observations (25–29% in [12,36]). Out of 58 control individuals, we had only a single homozygous TT horse. This underlines the importance of well-defined limits and accurate phenotyping for the control group horses when studying late-onset diseases to prevent biased results through horses wrongly considered tumour-free.

The manifestation of the DDB2-T variant was proposed as recessive. But, for our dataset, a model with genotypes as a fixed categorical effect fits best. Overall, we could prove that in comparison to homozygous DDB2 CC individuals, homozygous DDB2-T allele carriers (7.15 times the risk/OR 247) and heterozygous CT Haflingers (2.69 times the risk/OR 3.67) have a higher risk for developing an OSCC. Several recent case studies describe patients with only one variant of a biallelic Mendelian disease who display an intermediate phenotype somewhere on the continuum between affected, symptomatic patients and unaffected individuals [58]. For the increased risk for OSCC in heterozygous Haflingers, we can, at the moment, only speculate on the underlying cause. The observed incomplete dominant effect may result from haploinsufficiency (where a single functional allele is not producing sufficient gene product for proper function) or due to a dominant negative effect (which occurs when the expression of the mutant protein interferes with the activity of a wildtype protein). Reasonable other explanations are the complex genetic basis, as more genetic loci are involved (oligogenic/polygenic) and/or the composite effect of environmental modifiers.

Among the patients at Vetmeduni, the age of OSCC onset was later in Warmbloods than in Haflingers. This finding is in agreement with previous studies [5,16] that also reported an earlier age at diagnosis in Haflinger compared to other breeds. We need to mention that our mean age (15.77 ± 5.45 for Warmbloods and 13.3 ± 5.68 for Haflinger) was later than the estimates in the above-mentioned studies (13 years for Warmbloods; 8–10 years for Haflinger). The age of onset herein was set with diagnosis at the clinic. As patients show up at different (often progressive/late) stages of tumour development, they were treated for a certain period before, and the age of onset had most likely been earlier. This explains the shift towards older horses in our dataset. However, we assume a similar shift in all breed and age groups. As the DDB2 risk allele was not detected in Warmbloods (Table 2), we questioned whether the age differences can be attributed to the DDB2 genotype in Haflingers. In agreement with previous findings [16], we observed that numerous Haflingers with DDB2 genotype TT are diagnosed with OSCC at a young age. However, the effect is not statistically significant due to the low number of CC and CT cases in our dataset (Figure 4b). Also, in agreement with previous studies, we found no differences between Haflinger DDB2 genotypes with regard to sex [16].

Besides the Haflinger, the DDB2 risk allele has been previously found in other breeds, especially in draft horses, Appaloosas, some Warmbloods, and Rocky Mountain horses [12,59]. We observed an increased risk for OSCC and the DDB2 risk allele for the first time in an alpine draft breed—the Noriker. Our sample size for Noriker with OSCC was seven individuals, which was relatively small, but four out of those were homozygous DDB2 risk allele carriers. The Noriker is a common draft horse breed in Austria and is genetically closely related to the Haflinger due to their shared ancestry [60]. The segregation of the DDB2-T allele in the Noriker is not unexpected, given the relatedness of the two breeds. Also, the glycogen synthase 1 (GYS1) mutation (associated with Polysaccharide Storage Myopathy Type 1) has been detected in both breeds [61,62]. Moreover, comprehensive genotyping to assess the allele frequency of the DDB2 risk allele in the Noriker should be conducted together with a clinical investigation to evaluate the penetrance and, consequently, the relevance of the allele in this breed. Norikers and Haflingers are herded under similar environmental conditions, in particular, the common practice of alpine pastures during summer. While Haflingers are fixed for the chestnut colour, seven different coat colours are accepted in the Noriker breed [63]. Among the OSCC patients, four (two DDB2-TT, one CT, one CC) were chestnut/and or tiger, while only one was bay (DDB2-TT) and one roan (DDB2 CT). This observation again leads to the confounding impact of the pigmentation on the development of OSCCs. The Noriker breed, with a variety of coat colours, could be a suitable model to study the effect of photoprotectivity from pigment further [12].

Besides OSCCs, Haflingers are also prone to develop other skin tumours, like SCCs and ESs. A significant association between the DDB2 variant and SCCs at localisations other than the eye has not been detected yet. But, the single study was based on only eight horses with oral SCC (out of which only two were Haflingers) and 73 horses with urogenital SCCs (no Haflinger included) [16].

Here, we determined the DDB2 genotype in patients with SCCs on the head and neck or the genital regions. We included Haflingers and Norikers (because the allele is segregating in these breeds), Warmbloods (because of the large number of patients) and Icelandic horses (because of their high incidence of genital SCC, as shown in Figure 1). Out of the 55 horses with SCCs at localisations other than the eye, only one Haflinger was identified as homozygous for the DDB2 risk allele. The various tumours that had developed in this horse already at the age of 9 years (Section 3.4) classify this horse as a unique cancer patient. Together with the unclear lesion in the eye, the clinical picture could be attributed to its DDB2-TT genotype. Three horses, two Haflingers and one Warmblood, with an SCC in the head and neck region, were found to be heterozygous carriers.

We could not detect the DDB2-T allele in any patient with genital SCC. Hence, we conclude that the causes of genital SCCs must lie in infectious agents such as EcPV [13,21,22,23,24] and other predisposing factors such as breed, coat colour, castration, and hygiene status [64] and not in the DDB2 genotype of the horse. Last, we analysed the impact of the DDB2 risk allele on the development of the most common skin tumour in equids, the ES. We focused in our Sarcoid screen again on the two breeds in which the DDB2 risk allele segregates, Haflinger and Noriker. No horse was a homozygous carrier of the DDB2 risk allele. We deduce that the DDB2 risk allele plays no role in the etiopathology of SCC or ESs in localisations other than the eye.

Here, we provide further evidence for the impact of the DDB2 mutation on the development of OSCC. However, not all the cases show the mutation. In the future, the impact of other genomic regions, such as the region of homozygosity on Chr3 containing the UV-stimulated scaffold protein A (UVSSA) [65], should also be considered. Our sampling is based on horses coming to the clinic and, therefore, skewed towards sick horses. In addition, broad field examinations should be conducted to evaluate the penetrance of the allele in a more unbiased way. Moreover, Haflinger and Noriker, two breeds with closed studbooks and shared ancestry (but the Noriker representing a variety of coat colours), provide a perfect environment to investigate the impact of the different factors leading to OSCC.

## 5. Conclusions

The results from this study confirm the clinical consequences of the DDB2 risk variant and underline its severity with regard to animal health. Knowing the DDB2 allele status of their horses is essential for breeders, veterinarians, and private owners. In Haflingers and Norikers, regular veterinary eye checks for early detection of OSCC should be conducted. The DDB2 genotype should be taken into account in mating strategies in the Haflinger, and further investigation is necessary in the Noriker breed.

## Figures and Tables

**Table 1:** DDB2 genotyping results from Noriker and Haflinger FFPE samples before and after UDG treatment.

DDB2 Genotype Prior FFPE Treatment	Number of Samples/Genotype After UDG Treatment Confirmed	Mean (min/max) Years in Paraffin
CC	16/16	9.43 (2/17)
CT	12/12	8.75 (3/17)
TT	33/33	9.03 (1/19)

**Table 2:** DDB2 genotyping results in 144 cases and 58 Haflinger controls.

Group	Genotype	Haflinger	Noriker	Warmblood	Icelandic Horse
OSCC	CC	6	1	14	
	CT	11	2	-	n.e.
	TT	39	4	-	
SCC *head and neck*	CC	4	1	10	4
	CT	2	-	1	-
	TT	1	-	-	-
SCC *genital*	CC	5		15	9
	CT	-	n.e.	-	-
	TT	-		-	-
ES	CC	10	2		
	CT	1	2	n.e.	n.e.
	TT	-	-		
Tumour free	CC	38			
	CT	19	n.e.	n.e.	n.e.
	TT	1			

n.e. = no sample examined.

## Data Availability

All data are included in this submission.

## Supplementary Materials

The following supporting information can be downloaded at https://www.mdpi.com/article/10.3390/ani15070911/s1, Table S1: Genotyping sample information Table; Table S2: Information on Breeds of Patients presented to the Centre for Equine Health and Research of the University of Vetmeduni Vienna 2002–2022.
